# Effects of Trophic Acclimation on Growth and Expression Profiles of Genes Encoding Enzymes of Primary Metabolism and Plastid Transporters of *Chlamydomonas reinhardtii*

**DOI:** 10.3390/life13061398

**Published:** 2023-06-15

**Authors:** Roman K. Puzanskiy, Daria A. Romanyuk, Anastasia A. Kirpichnikova, Maria F. Shishova

**Affiliations:** 1 Laboratory of Analytical Phytochemistry, Komarov Botanical Institute of the Russian Academy of Sciences, St. Petersburg 197022, Russia; puzansky@yandex.ru; 2 Faculty of Biology, St. Petersburg State University, St. Petersburg 199034, Russia; 3 Department of Biotechnology, All-Russia Research Institute for Agricultural Microbiology, Pushkin, St. Petersburg 196608, Russia

**Keywords:** *Chlamydomonas reinhardtii*, autotrophy, adaptation, acclimation, cultivation, microalgae, mixotrophy

## Abstract

In this paper, the effect of prolonged trophic acclimation on the subsequent growth of *Chlamydomonas reinhardtii* batch cultures was studied. The mixotrophic (light + acetate) acclimation stimulated subsequent growth at both mixotrophy and autotrophy conditions and altered the expression profile of genes encoding enzymes of primary metabolism and plastid transporters. Besides the trophic effect, the influence of Chlamydomonas culture growth stage on gene expression was determined. Under mixotrophic conditions, this effect was most pronounced in the first half of the exponential growth with partial retention of the previous acclimation period traits. The autotrophy acclimation effect was more complex and its significance was enhanced at the end of the growth and in the stationary phase.

## 1. Introduction

*Chlamydomonas reinhardtii* is a unicellular green alga capable of assimilating exogenous acetate [1]. The ability to consume exogenous organic substrates is an important adaptive trait and increases the number of ecotypes inhabited by this alga. The accessibility of energy and carbon determines the predominance of the autotrophic and/or heterotrophic machinery of Chlamydomonas metabolism, as well as the redistribution of resources between the processes of cell division, growth, and deposition. It defines the balance between synthesis and catabolism in algal cells [2,3]. The effect of trophic conditions on *C. reinhardtii* growth and metabolism has been studied at genetic, biochemical, and physiological levels through newly developed transcriptomic [4,5,6], proteomic [7,8,9], and metabolomic [7,10,11] approaches. Nevertheless, in comparison with other types of adaptation strategies [12,13], many processes involved in tropic adaptation are still far from known.

Limited studies have indicated that acclimation to previous trophic conditions affect further algae growth strategies, for example, determination of the lag phase duration. After *Cyanidium caldarium* transfer from heterotrophy to autotrophy, a lag phase of 10–15 h was observed, in contrast with mixo and heterotrophic conditions [14]. In the case of *Galdieria sulfuraria*, the lag phase duration varied greatly because of the conditions during the transition from auto to heterotrophy and the type of substrate used [15]. In *C. sorokiniana*, changes from auto to the mixotrophic conditions induced the appearance of a lag phase, the time of which ranged from several hours to 1.5 days and that positively depended on the concentration of acetate [16]. The diversity of responses of green algae across a gradient of phototrophic, mixotrophic, and heterotrophic conditions was recently discovered [17]. The modulation of the lag phase duration is due to the launch of new mechanisms responsible for the uptake and assimilation of new substrates, and could reflect traits of algae metabolism and physiology.

The necessity of metabolic adaptation to a new growing mode has been demonstrated in several studies. For example, in the absence of pre-incubation, glucose uptake by *Chlorella vulgaris* cells started 90 min after its addition, regardless of lighting, or immediately if the culture was pre-adapted to glucose [18]. Pre-incubation (2 days) of psychrophilic *Chlamydomonas* sp. with glucose in darkness resulted in double increases in the uptake of carbohydrates [19]. Trophic acclimation was shown to be substrate specific. The culture of *Euglena gracilis* Z, acclimated to glucose, reached a lower cell density in media with butyrate in comparison with the culture with glucose, which continued to grow [20]. The acclimation of *C. coldarium* in glutamate-containing media caused a five-fold increase in the extrusion of ammonium during further growth with the same amino acid in comparison with that after being transferred to media with other amino acids. Apparently, pre-adaptation required the induction of protein synthesis and an alteration in enzyme activity. Aminotransferases and enzymes of the catabolism were upregulated, but the dehydrogenase activity decreased [21]. Some additional data are in agreement with the importance of pre-acclimation for further microalgae development. For example, after the transfer of Chlamydomonas to darkness, the expression of the *cabII-1* gene decreased. The shorter the previous illumination period, the faster the decrease in expression occurred [22]. The acclimation was also found to be significant for complex dynamic processes. Complicated diurnal metabolic rearrangements coordinated with alterations in the gene expression were determined in Clamydomonas under light/dark circles [23]. Circadian rhythms of starch metabolism, developed under periodic illumination (at least 4 days), were retained by *C. reinhardtii* cells (at least a week) after transfer to darkness or to constant illumination [24].

**Figure 1:**
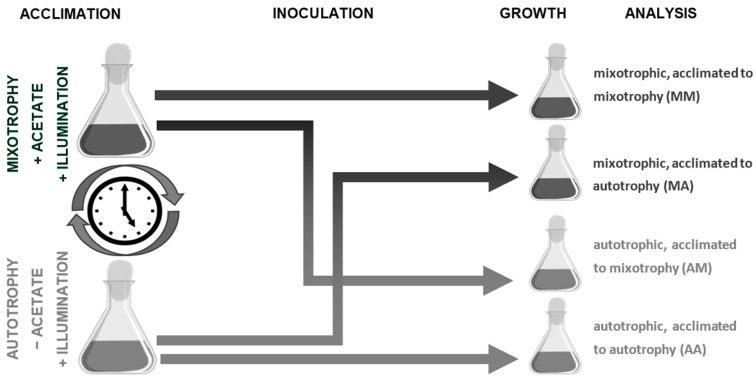
Experiment setup.

The purpose of this study was to distinguish the role of prolonged trophic acclimation of batch *C. reinhardtii* cultures to mixotrophic or autotrophic conditions for the determination of the subsequent growth rate and development. Focus was given to identifying the link between two alterations: the change in culture medium to a new one and caused by the transfer of cultures, acclimated to auto or mixotrophic conditions, to a new nutrition regime (Figure 1). As a test system, the expression of the genes encoding the enzymes of the primary metabolism and plastid transporters were used (Table S1). These genes were selected in our previous studies [6,25,26]. The results reveal the significant role of these genes in the acclimation of batch *C. reinhardtii* cultures to auto and mixotrophic conditions.

## 2. Materials and Methods

### 2.1. Strains and Cell Culturing

Cultures of *Chlamydomonas reinhardtii* P.A. Dangeard 1888 CC-124 were kindly donated by Dr. E.M. Chekunova, Department of Genetics and Biotechnology, Faculty of Biology, St. Petersburg State University. Axenic cultures were maintained under mixotrophic conditions on TAP (Tris–Acetate–Phosphate) medium [27] or autotrophic conditions on TM (Tris Minimal, without acetate) medium in half-filled 500 mL cone glass flasks, corked with cotton stoppers. The cultures were grown under constant illumination with fluorescent lamps with an intensity of 42 µmol m^−2^ s^−1^ photons PAR. To determine the effect of acclimation (Figure 1), autotrophic (AA), and mixotrophic (MM) cultures were maintained in this mode for at least a year, which caused acclimation to these trophic conditions. Furthermore, some of these cultures were transferred to other nutritional conditions (i.e., cultures acclimated to autotrophy—to mixotrophic (AM) or mixotrophic—to autotrophic (MA)). The initial cell density was equalized. Three independently grown cultures corresponded to three biological replications. Samples were taken four times during culture development. At the beginning of exponential growth (first DAI (day after inoculation) for mixotrophic, third DAI for autotrophic), its middle (second DAI for mixotrophic, sixth DAI for autotrophic), the second half—completion of exponential growth (fourth DAI for mixotrophic and twelfth DAI for autotrophic), and in the stationary phase (sixth DAI for mixotrophic and twentieth DAI for autotrophic).

### 2.2. Transcription Analysis

Transcription analysis was performed as described earlier [6,26]. In brief: 10^7^–10^8^ of cells were collected by gentle centrifugation (5 min, 3000× *g*, 4 °C). The pellet was resuspended in 1 mL of PureZOL™ (PureZOL ™, Bio-Rad Laboratories, Hercules, CA, USA) and shacked vigorously. The samples were frozen and stored at −80 °C. After thawing, the samples were incubated at room temperature for 10 min. Then, the homogenate was centrifugated at 12,000× *g* for 10 min at 4 °C to discard the cell debris. Homogenate was supplemented with 0.2 mL of chloroform, shacked vigorously for 15–20 s, and incubated for 5 min at room temperature with stirring. The samples were then centrifuged at 4 °C during 15 min at 12,500× *g* and the aqueous phase was collected. Then 0.5 mL of isopropanol was added and the samples were incubated for 10 min at room temperature. The samples were centrifuged for 10 min at 4 °C and 12,500× *g* and the supernatant was removed. The pellet was washed 75% ethanol, dried, and dissolved in 20–25 µL of deionized DEPC water. The quality and quantity of the isolated RNA were determined on a NanoDrop 1000 spectrophotometer (Thermo Fisher Scientific, Waltham, MA, USA). The RNA samples were DNase I (Thermo Fisher Scientific) treated to avoid genomic DNA contamination. cDNA was synthesized with M-MuLV reverse transcriptase (Thermo Fisher Scientific), applying RiboLock RNase inhibitor (Thermo Fisher Scientific). The sequences of primers are given in Table S1. The primers were ordered from BioBeagle (St. Petersburg, Russia). qRT-PCR was performed with SYBR Green I intercalating dye (Syntol, Moscow, Russia) on a CFX96 Real-Time PCR Detection System with a C1000 thermal cycler (Bio-Rad Laboratories, Hercules, CA, USA) using the following program: 7 min at 95 °C, then 40 cycles at 95 °C for 15 s, and at 60 °C for 50 s. The hardware of the Research Park of the Center for Molecular and Cell Technologies (St. Petersburg State University) was used. *RPL19* encoding ribosomal protein L19 was used as a reference gene. The relative expression was calculated according to 2^−ΔΔCt^ method.

### 2.3. Data Analysis

The analysis was carried out in the R environment [28]. The principal component analysis (PCA) method was performed using the *pcaMethods* package [29]. The data were logarithmized and standardized. The number of components was selected by cumulative explained variance and eigenvalues (Figures S1a,b and S2a,b). The differences in the profiles caused by the acclimation were tested. PERMANOVA (Permutational Multivariate Analysis of Variance) was applied using Euclidean distances in the space of score plot [30]. For this purpose, *adonis2* function from the *vegan* package was used. 3D plots were made with *scatterplot3d* package [31]. The Euclidean distance in the space of the first three principal components was calculated. OPLS-DA was carried out in the *ropls* package [32]. The reliability of the models was assessed by the R^2^Y, Q^2^Y values and their probabilities after permutation. VIP (Variable Importance in Projection) estimates the relationship between the variable and a class difference. Variables with VIP > 1 were considered to be reliably related to class differences. The Spearman correlation coefficient (rho) was run to determine the similarity of loadings of different models. Hierarchical cluster analysis was performed using the Pearson distance (1 − r). Ward’s method was followed to aggregate the clusters. A scree plot was used to determine the number of clusters. To compare the average values of culture densities, a t-test was applied. Metabolic network was visualized in Cytoscape [33].

## 3. Results

### 3.1. The Effect of Trophic Acclimation on the Growth Rate

The scheme of the experiment, which focused on elucidating the effect of long-term cultivation (at least a year), which we termed acclimation, on the further growth and gene expression of *C. reinhardtii* is shown in Figure 1. At first, the effect of trophic acclimation on cell density of batch auto and mixotrophic cultures of *C. reinhardtii* was analyzed. Pre-cultivation in the presence of acetate intensified the growth rate of cultures, regardless of the trophic conditions during the experiment (AM and MM variants). This effect was most obvious during the first half of the exponential phase (Table S2). After 2 days (the middle of the log phase), the density of MM cultures (CD = 1.13 ± 0.13 SD; CD—cell density of 10^6^ cells/mL; SD—standard deviation) exceeded that of the MA cultures (CD = 0.43 ± 0.14 SD) by almost three times (*p* = 0.0003). In the case of autotrophy, similarly, the AM density (CD = 0.79 ± 0.21 SD) was twice (*p* = 0.02) higher than in the AA variant (CD = 0.35 ± 0.14 SD) on the sixth DAI of culturing. It is important to emphasize that acclimation did not significantly affect the density of the culture in the stationary phase in the case of both autotrophic (CD_AA_ = 4.59 ± 0.65 CD_AM_ = 4.28 ± 0.54 *p* = 0.27) and mixotrophic cultures (CD_MM_ = 9.41 ± 0.95, CD_MA_ = 8.46 ± 0.94, *p* = 0.27).

### 3.2. The Effect of Trophic Acclimation on the Expression Profiles of Mixotrophic Cultures

To elucidate the mechanisms involved in the effect of acclimation, the alterations in the expression of 32 genes encoding enzymes of primary metabolism and plastid transporters were studied.

**Figure 2:**
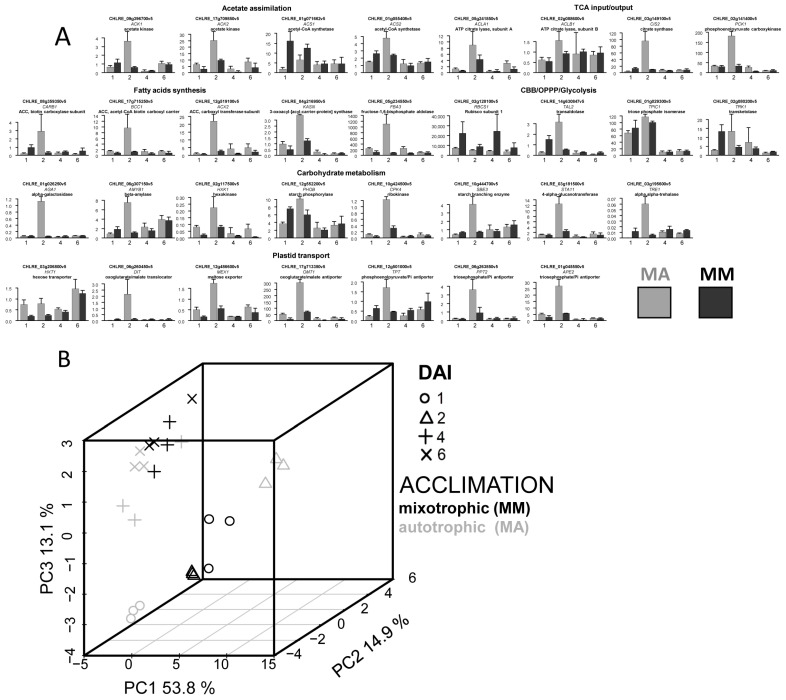
Expression profiles of the genes coding the enzymes of central metabolism and plastid transporters in *C. reinhardtii* mixotrophic cultures acclimated to mixotrophy (MM) or autotrophy (MA). (**A**) Relative gene expression; intervals—SD; abscissa—DAI (day after inoculation). (**B**) Score plot from the PCA of the expression profiles.

The relative expression is presented in Figure 2A (Table S3). It can be seen that the expression levels varied significantly during culturing. In most cases, the greatest changes in transcript accumulation were detected at the initial stages of culture development, especially in the MM variant. The obtained results indicate that a number of metabolic processes would change their intensity. Acetate assimilation, carbon metabolism, and lipid and starch metabolism could be of a special interest. The complexity of the detected changes increased, as they represent the sum of responses caused by the renewal of the environment and the replacement of the trophic status. It is quite difficult to distinguish the importance of both.

In order to reveal the distinction of the expression profiles detected after the changing trophic conditions, they were presented in a low-dimensional space. The results of the PCA (principal component analysis) are represented as a score plot (Figure 2B). The greatest divergence in cultures with different acclimations was observed on the first and second DAI. Consequently, acclimation had the maximum effect on the gene expression profile at the beginning of the mixotrophic culture development. PERMANOVA, in the space of the first three PC scores, showed a significant effect for DAI (*p* = 0.003) and the type of acclimation (*p* = 0.014). However, there was no statistically significant interaction between the factors (*p* = 0.7).

A more detailed PCA for each time point separately (first, second, fourth, and sixth DAI) also showed that the transcription profiles were grouped according to the conditions of trophic acclimation. The dependence was more pronounced on the first and second DAI (Figure S3). The differences in this case were associated with PC1 (FDR < 0.05; Table S5), except for the final time point which corresponded to the stationary phase.

**Figure 3:**
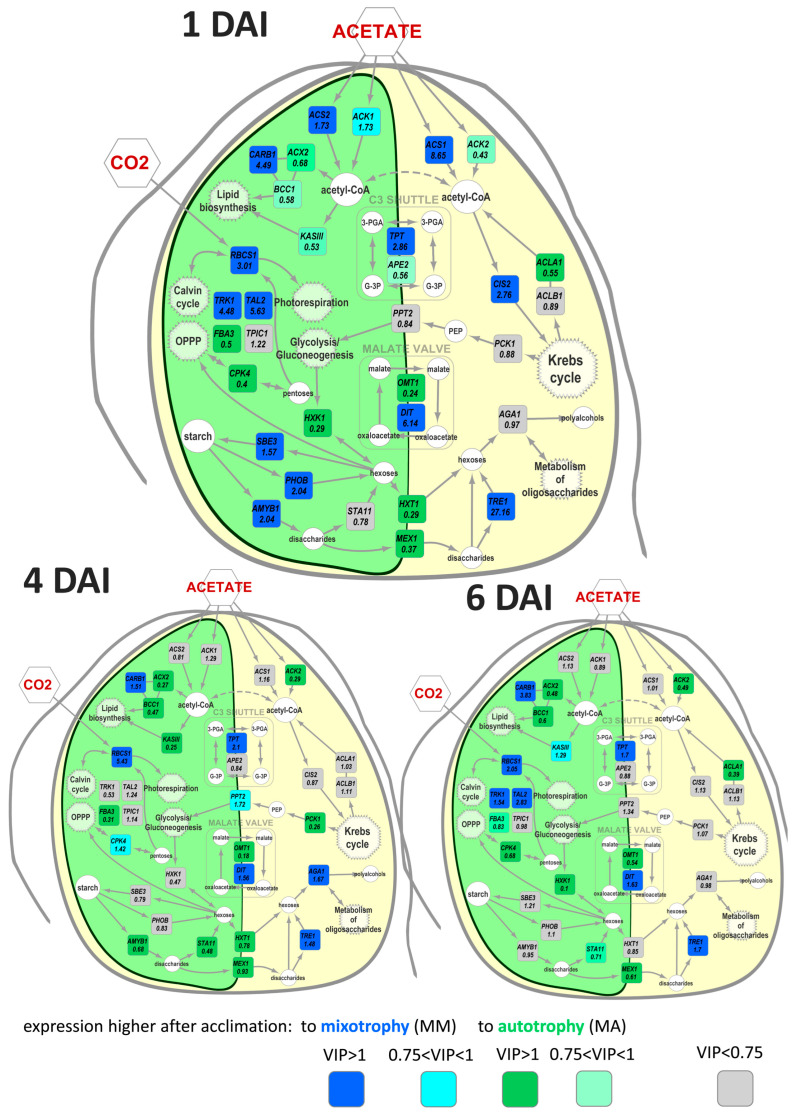
Visualization of differences in gene expressions determined by OPLS-DA: Numbers are fold changes MM/MA. DAI—day after inoculation.

To compare the expression profiles of MM and MA cultures for each time point, OPLS-DA models were developed (the parameters of the model are presented in Table 1). Higher percentages of variance associated with the predictive component were obtained for the beginning and middle of the exponential phase. Differently expressed genes (DEGs) were visualized in the simplified scheme of the Chlamydomonas metabolic pathways. The effect of acclimation was similar on the first, fourth, and sixth DAI in general (Figure 3), whereas in the middle of the growth phase (2nd DAI) this differed significantly and was reduced to a common pattern: the vast majority of genes that had the maximum expression on the second DAI in MM were characterized by a higher expression in MA. The exceptions were *ACS1* and *RBCS1* genes, which were more expressed in MM and at the beginning of growth.

In detail, at the beginning of the growth, MM cultures accumulated more transcripts of *ACS1* and *ACS2* genes encoding acetyl-CoA synthases, which are enzymes of acetate utilization. At the same time, acclimation to mixotrophy was associated with a higher expression of genes involved in carbohydrate fixation, including the *RBCS1* gene, which encodes the small Rubisco subunit. MM cells were characterized by a higher expression of genes encoding enzymes of the pentose phosphate pathway (PPP) such as transketalase (*TRK1*) and transaldolase (*TAL2*). Unlike *RBCS1*, differences in the expression of *TRK1* and *TAL2* disappeared at the end of the growth phase and reappeared in the stationary phase, but were less evident than at the beginning. At the early growth stage, higher transcript accumulation of the genes encoding the enzymes involved in starch metabolism, such as *AMYB1* (β-amylase), *PHOB* (starch phosphorylase), and *SBE3* (starch branching enzyme), was detected. *TRE1* (α,α-trehalase) was upregulated after acclimation to mixotrophy during the entire development.

The acclimation with acetate and its further presence in media (MM) had a different effect on the acetyl-CoA-carboxylase subunits. A higher level of expression was estimated for the *CARB1* gene encoding biotin carboxylase, while the genes of other two subunits *BCC1* (biotin-carboxyl-transferring protein) and *ACX2* (α subunit of carboxyl transferase) showed the opposite trend. The expression of the gene of another enzyme, directing acetyl groups in the synthesis of fatty acids *KASIII*, showed an increase in the growth phase of MM, but was kept at a higher level for most of the growth in the cells of the MA variant.

After acclimation to autotrophy, a more intense expression of genes encoding sugar kinases was observed in mixotrophic cultures (MA) for *HXK1* (hexokinase) and *CPK4* (ribokinase). The effects of acclimation on these two genes were less pronounced at the end of the growth phase. Acclimation to autotrophy positively regulated the expression of *FBA3*, but later it decreased at the stationary phase. In the MA variant, a higher accumulation of transcripts for genes encoding the plastid sugar transporters were distinguished. These transporters ensured the outflow of carbohydrates from the plastid—HXT1 (hexose transporter) and MEX1 (maltose exporter). This effect was more acute at the beginning and middle of the exponential phase.

**Figure 4:**
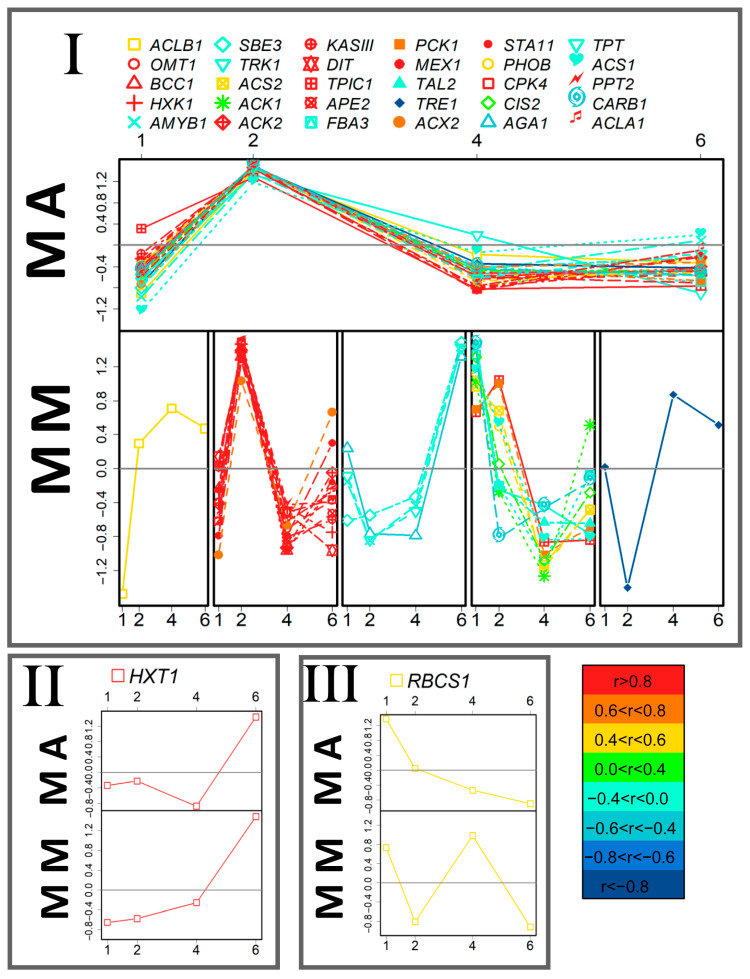
Comparative analysis of the patterns of the gene expression dynamics in mixotrophic cultures of *C. reinhardtii*. The genes were divided into three groups (I–III) as a result of PCA with distance measured as the Pearson correlation of the expression levels in the mixotrophic cultures acclimated to autotrophy (MA). For comparison, the dynamics patterns of the same genes in mixotrophic cultures acclimated to mixotrophy (MM) are presented (also clustered by the expression dynamics). Colors mark the level of correlation between MM and MA cultures. The data on the graphs are standardized. Abscissa—day after inoculation.

Further comparative analysis of the expression profiles alterations showed that in the case of both MM and MA cultures, it had common traits (Figure 4): most of the tested genes had a maximum expression on the second DAI. Another similarity was the decrease in the expression level of the most genes during the transition to the stationary phase. This dynamic was more pronounced in the MA variant (Figure 4). However, a number of genes, with a higher expression on the first DAI in MM, were characterized by a greater accumulation of transcription products than in the MA variant. This indicates that these genes reached their expression maximum in MA later than in MM.

**Figure 5:**
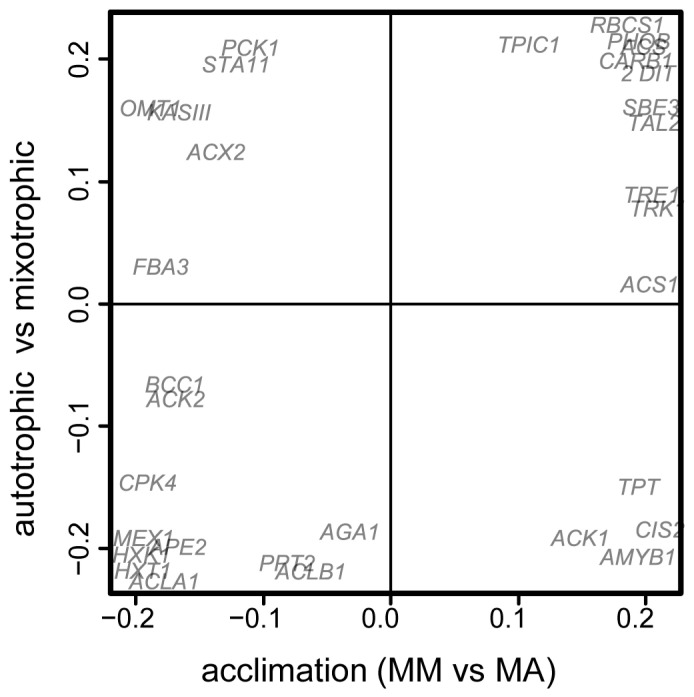
Comparative analysis of the effect of trophic conditions and acclimation on mixotrophic *C. reinhardtii* cultures. Scatter plot in the space of factor loadings of predictive components from two OPLS-DA models: the first (abscissa)—for comparison of mixotrophic cultures acclimated to auto (MA) and mixotrophy (MM); the second model (ordinate)—for comparison of mixotrophic (MM, 1st DAI) and autotrophic (AA, 3rd DAI) cultures at the beginning of growth. Positive values correspond to a higher level of expression during mixotrophy and acclimation to it.

The next step of our study was to analyze, to what degree, the differences in mixotrophic cells, acclimated to autotrophy, were the result of the acquisition of trophic specialization after previous autotrophic cultivation. For this purpose, a SUS plot was created (Figure 5): scattering of genes in the space of loadings of predictive components from OPLS-DA for the comparison of MM and MA on the first DAI (abscissa) and auto and mixotrophic cultures during the beginning of exponential growth (first day for MM and third for AA). A significant number (22 out of 32) of genes had the same signs of loading, which significantly differed from randomness (*p* = 0.025). The correlation in loadings was reliable (rho = 0.43, *p* = 0.015), but not strong. At the later stages, this correlation disappeared. It is assumed that even after the transition to mixotrophy, at least at the initial stage of cultivation, the metabolic traits of autotrophic cultures remained.

### 3.3. The Effect of Trophic Acclimation on the Expression Profiles of Autotrophic Cultures

**Figure 6:**
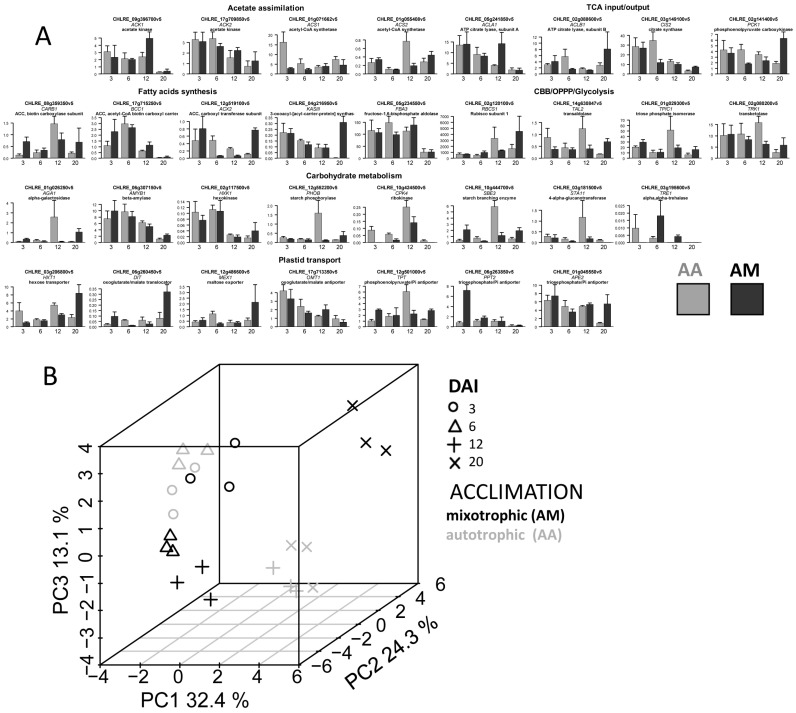
Expression profiles of genes encoding enzymes of central metabolism and plastid transporters in *C. reinhardtii* autotrophic cultures acclimated to mixotrophy (AM) or autotrophy (AA). (**A**) Relative expression, intervals—SD; abscissa—DAI (day after inoculation). (**B**) Score plot from PCA of expression profiles.

The alterations in the relative expression of genes in autotrophic cultures acclimated to autotrophy (AA) or mixotrophy (AM) are represented in Figure 6A (Table S3). As in the case of mixotrophic cultures (Figure 2B), transcript accumulation varied due to the process of acclimation and culture stage.

Further PCA application did not show a distinct global grouping of expression profiles depending on acclimation (Figure 6B and Figure S2). However, clustering of samples was observed according to the age of the Chlamydomonas culture. Thus, the differences within each age group (according to DAI), depending on acclimation, were also discovered. PERMANOVA on the scores of the first four PCs, also showed a significant effect of culture age (*p* = 0.001). Unlike the variant with acetate mixotrophy, there was no independent significant effect of acclimation in autotrophic conditions (*p* = 0.4), whereas a significant interaction for age and acclimation (*p* = 0.008) was observed. It can be concluded that acclimation affects the dynamics of expression profiles during culture ageing.

An analysis of each time point separately, using PCA, revealed that the samples were grouped according to the type of trophic acclimation that was opposite to the variant with mixotrophic cultures. The distinction was clearer at the last two time points (Figure S4). Nevertheless, the differences in PC1 values for AA and AM cultures at each time point were statistically significant (Table S2).

**Figure 7:**
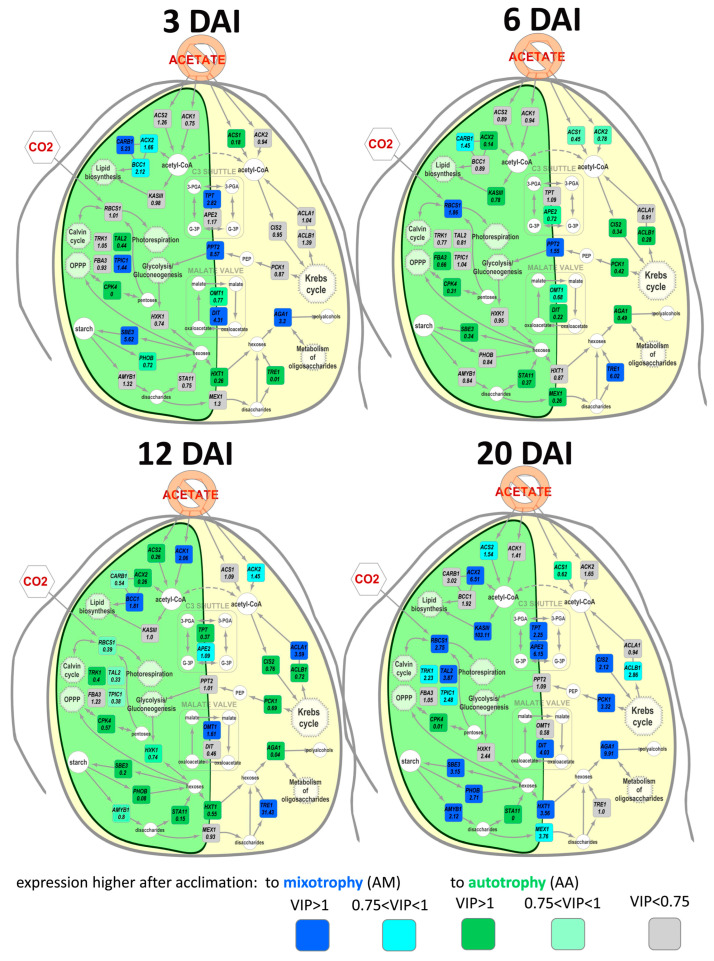
Visualization of the differences in gene expressions determined by OPLS-DA: Numbers are fold changes MM/AA. DAI—day after inoculation.

OPLS-DA models were made for each of the studied time points. The parameters of the models are given in Table 1. An essential part of variance associated with the predictive component was observed at the end of the exponential and in the stationary phase. The effect of acclimation on autotrophy at different DAIs is demonstrated in Figure 7.

At the early exponential phase (third DAI) in AM cultures, the expression level of the genes encoding acetyl-CoA carboxylase subunits *BCC1* and *CARB1* exceeded that in the AA variant, as well as *TPIC1* encoding triose phosphate isomerase, possibly involved in the supply of intermediates for lipid synthesis pathways. Similarly, the expression of the *PPT2*, *DIT*, and *TPT* genes, which encode plastid transporters, was higher. In turn, in AA, during this period, transcripts of the *CPK4*, *ACS1*, *TAL2*, and *HXT1* genes accumulated more intensively. These genes, with the exception of *ACS1*, are associated with carbohydrate metabolism, which probably proceeded at a higher extent under autotrophic conditions and after acclimation to autotrophy.

In the mid-growth stage (sixth DAI), the pattern of differences between AA and AM changed drastically. The number of genes that had an upturned expression after acclimation to autotrophy increased. This acclimation continued to stimulate the transcript accumulation of the *CPK4* and, to a lesser extent, *ACS1* genes. An increase in expression was recorded for the *ACX2* and *KASIII* genes encoding the enzymes involved in the synthesis of fatty acids. Furthermore, in AA at sixth DAI, higher levels of expression for *FBA3*, *SBE3*, *MEX1*, and *AGA1* encoding fructose bisphosphate aldolase, starch branching enzyme, plastid maltose exporter and α-galactosidase, respectively, were detected. Stimulation by acclimation to autotrophy of the *STA11* gene activity associated with the decomposition of disaccharides in the plastid, opposite to the repression of the TRE1 cytosolic enzyme, indicated the redistribution of this process between compartments throughout acclimation. In cultures acclimated to autotrophy, a higher level of expression of the components of the malate valve *DIT* and, to a lesser extent, *OMT1* was observed. In AA cultures, the level of expression of genes associated with the input/output of carbon into/out of the Krebs cycle (*CIS2*, *ACLB1*, and *PCK1*) was higher. AM was still characterized by a more intense expression of *PPT2*. During this period, the expression level of *RBCS1* and *TRE1* was also higher in the AM cultures.

At the end of the growth period (twelfth DAI) in the AA variant, the number of highly expressed genes exceeded that in the AM cultures and, in general, the discovered alterations were similar to those at the previous time point. Acclimation to autotrophy continued to facilitate a higher level of gene expression associated with the input and output of carbon from the Krebs cycle. AA culture cells still expressed more *CPK4*. Interestingly, acclimation during this period affected the expression of genes encoding the enzymes involved in the metabolism of acetyl groups in plastid. The expression of *ACS1* was more intensive in AA, but *ACK1* was more intense in the AM cultures. In the case of acetyl-CoA-carboxylase and isocitrate lyase, unexpectedly, the effect of acclimation on the expression of the genes encoding the subunits of these enzymes was multidirectional. In turn, the transcript accumulation for the genes encoding the enzymes of carbohydrate metabolism and plastid transporters was more developed in the AM cultures.

In the stationary phase (twentieth DAI), the obtained profile was different. The pool of genes with an intensified expression was larger after acclimation to mixotrophy. This acclimation stimulated the accumulation of transcripts of enzyme genes associated with carbon fixation, starch metabolism, carbon input, and output from the Krebs cycle and plastid transporters. Acclimation to autotrophy contributed to a higher level of expression for genes associated with PPP and disaccharide metabolism in the plastid.

**Figure 8:**
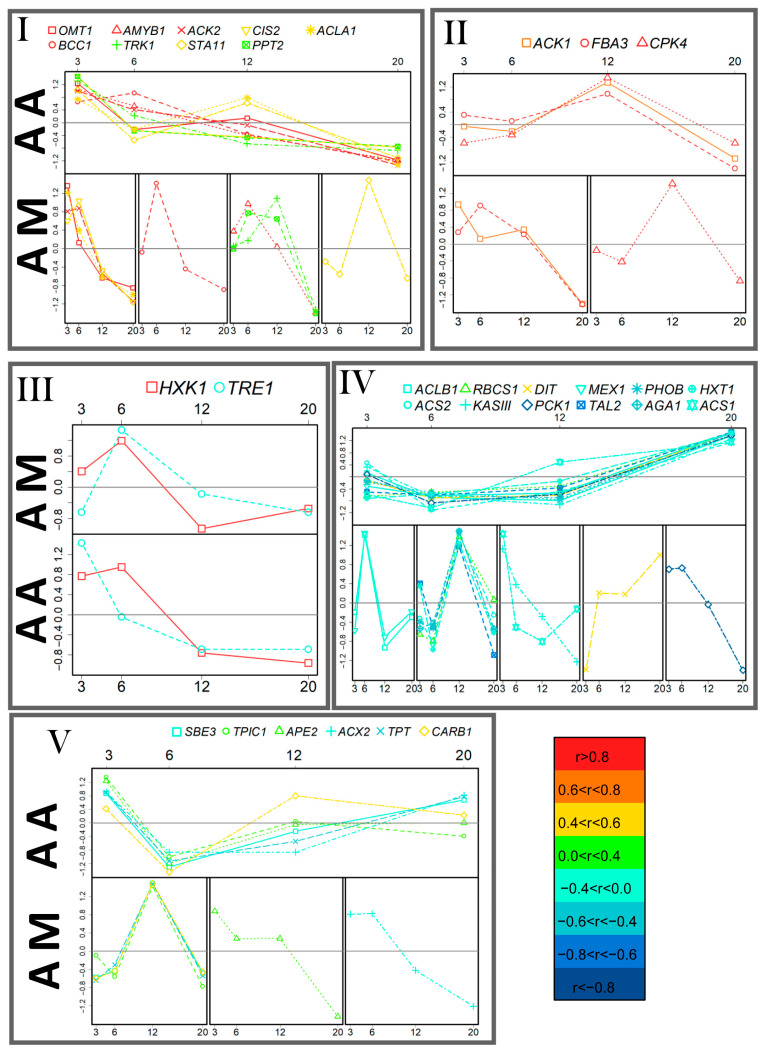
Comparative analysis of the patterns of the gene expression dynamics in autotrophic cultures of *C. reinhardtii*. The genes are divided into five groups (I–V) as a result of PCA with distance measured as the Pearson correlation of expression levels in autotrophic cultures acclimated to mixotrophy (AM). For comparison, the dynamics patterns of the same genes in autotrophic cultures acclimated to autotrophy (AA) are presented (also clustered by expression dynamics). Colors mark the level of correlation between AA and AM cultures. The data on the graphs are standardized. Abscissa—day after inoculation.

Autotrophic cultures acclimated to mixotrophy were characterized by dynamic changes in the expression profile during cell growth. To identify the main patterns of the dynamics of expression levels in AM, a cluster analysis was performed using correlation as a measure of proximity. The results of the comparison of AA and AM expression patterns are shown in Figure 8. Chlamydomonas cells were characterized by a decrease in the expression level of a large number of genes during the aging of the culture and transition to the stationary phase (clusters I–III) in both variants. Another important trait of alteration in the expression level in AM cultures was the appearance of a large group of genes for which the expression increased with time (clusters IV and V).

**Figure 9:**
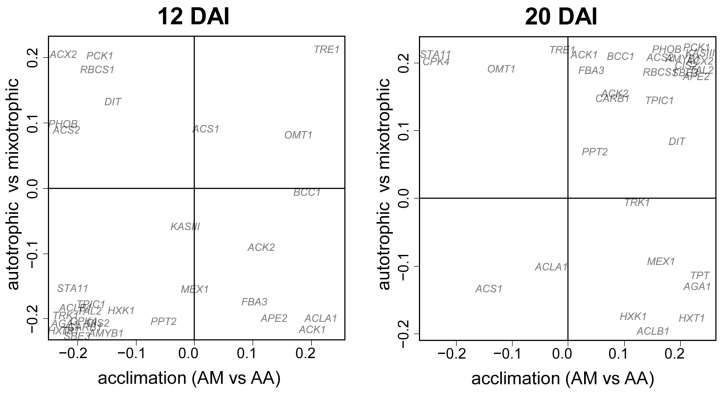
Comparative analysis of the effect of trophic conditions and acclimation on autotrophic *C. reinhardtii* cultures. Scatter plot in the space of factor loadings of the predictive component from two OPLS-DA models: the first (abscissa)—for comparison of autotrophic cultures acclimated to auto (AA) and mixotrophy (AM); the second model (ordinate)—for comparison of mixotrophic (MM, fourth and sixth DAI) and autotrophic (AA, twelfth and twentieth DAI) cultures at the end of the growth and stationary phases. Positive values correspond to a higher level of expression during mixotrophy and acclimation to it.

Then, a comparative analysis of the differences in the expression profiles of autotrophic cultures acclimated to auto and mixotrophy and the differences in auto and mixotrophic cultures was carried out. Figure 9 shows SUS plot in the space of loadings of predictive components from OPLS-DA models for comparison of autotrophic cultures acclimated to auto and mixotrophy at the twelfth and twentieth DAI (abscissa) and for the classification of auto and mixotrophic cultures in the same growth phases. Unlike mixotrophic cultures, no correlation between loadings was observed. However, in both cases, about half of the genes were grouped in one corner. At the end of the growth phase (twelfth DAI), this was an angle with negative load values, which corresponded to the repression of transcription both in mixotrophy and in the acclimation of autotrophic cultures to mixotrophy. Then, it changed to the opposite. The genes were grouped in the upper right corner, corresponding to a higher expression in mixotrophic cultures compared with autotrophic and AM cultures compared with AA.

## 4. Discussion

Microalgae, similar to other living beings, adjust their physiology and metabolism for better survival. Metabolic acclimation to environment generally requires time and activation of the cell resources. Alga metabolism differs significantly under autotrophy and mixotrophy. The latter is supposed to be a mutable equilibrium between autotrophy and exogenous organic uptake. Appeared biochemical trophic adjustments include complex alterations in the various pathways of central metabolism such as carbon fixation, sugar metabolism, TCA, and lipid and starch deposition [2,7,10,34,35]. These alterations are closely related to a shift in the expression of genes encoding the involved enzymes [4,6,36], which results in a redirection in metabolic fluxes [27,37,38]. The assumption is that current algae growth and metabolism would have the effect of previous trophic conditions. This investigation (Figure 1) clarified the effect of prolonged acclimation to certain trophic conditions (auto or mixotrophic) on the subsequent growth and development of batch *C. reinhardtii* cultures with different sources of carbon and energy.

The transition between different forms of trophic conditions is accompanied with unequal physiological and biochemical responses, including alteration in the expression of genes involved in resources utilization. Predominantly, it concerns the genes of acetate assimilation, which is appropriate to mixotrophy. The acetate synthases *ACS1, 2* genes had a much higher expression in the cells previously cultivated under mixotrophic conditions in comparison with the autotrophic ones (Figure 3). These genes encode key enzymes of acetate utilization [3] and its upregulation is a trait of mixotrophy [8]. Acetyl groups provide algae cells with energy through the TCA cycle or due to the glyoxylate cycle [2,39]. *CIS1* also exhibited a higher expression in the MM variant in comparison with MA at the beginning of Chlamydomonas growth. This gene codes citrate synthase 1, which directs acetyl groups to TCA. This pathway competes with direction acetyl groups to lipid biosynthesis and lowering the expression of CIS1 resulted in higher lipid accumulation [40]. At the same time, the expression of *ACLA* coding citrate lyase, which directs acetyl groups from TCA, was downregulated. This combination implemented pumping acetyl groups into the TCA/glyoxylate cycle. In summary, when acclimated to mixotrophy, the cells acetate assimilation mechanisms additionally upregulated from the very beginning, which presumably provides a faster activation of metabolism and a faster growth start. 

On the other hand, in mixotrophic cultures of Chlamydomonas, photosynthetic activity is also maintained at a high level [8,10,41]. In addition, with aging and acetate depletion, the metabolism of cells of mixotrophic batch cultures shifts towards autotrophy [10]. Thereby, the transition from mixotrophy to autotrophy does not require the reactivation of metabolic pathways associated with the autotrophic type of nutrition, already since they are already on function. The evidence is the expression of some genes involved in carbon fixation, including *RBCS1*, in mixotrophic cultures [4]. In our experiments (Figure 3), Chlamydomonas cells of the MM variant had more active genes from the “photosynthetic block” of metabolism in comparison with the MA variant. Therefore, algal cells acclimatized to mixotrophy presumably had an advantage in the implementation of photosynthesis due to the previous cultivation, which resulted in more active growth with the current nutrition. This effect was less pronounced after acclimatization to autotrophy; the determined expression of *RBCS1* was higher in AM compared with AA at later periods: in the middle of growth and in the stationary phase (Figure 7). 

Photosynthetically fixed carbon could potentially be oxidized through glycolysis or OPPP for energy supply. Carbohydrate kinases play a key role in routing sugars in a breakdown scenario. Our data show that acclimation to autotrophic conditions downregulated the expression of genes encoding sugar kinases *HXK1* and *CPK4*, which is a sign of sugar catabolism repression (Figure 3 and Figure 7). Together with the upregulation of genes related to carbon fixation, this may result in more active biomass accumulation in MM and AM cultures.

Alterations in energy and carbon supply are closely associated with further redistribution of resources between active metabolism and storage. One of the possible pathways for the carbon deposition is the incorporation of an acetyl group to fatty acids and further to lipids [2]. During mixotrophic acclimation, the elevation in the expression of *CARB1* encoding one of the subunits of the enzyme responsible for the synthesis of fatty acids was determined, while the genes encoding other subunits did not show the same transcripts accumulation. Such an unequal expression of the genes encoding different acetyl-CoA-carboxylase subunits was observed earlier [26,42,43,44]. This is likely a mechanism of enzyme activity regulation due to specific subunit properties. 

In turn, the genes of starch and oligosaccharides metabolism were also regulated during acclimation. Close cross interaction between lipid and starch storage through a partially common carbon pool was shown recently in *C. reinhardtii* [45]. Under mixotrophy at the first DAI, the MM cells showed a significantly higher expression of genes coding the enzymes both synthesizing (*SBE3*) and degrading starch (*AMYB*, *PHOB*, and *TRE1*). Under autotrophic conditions (AM), *SBE3* transcripts were more intensively accumulated at the beginning of growth (Figure 7). The theory is that upregulation of these genes is a result of intensive storage of carbohydrates due to the higher photosynthetic rate at mixotrophic cultivation.

The importance of different transport systems in eukaryotic cell functional activity is widely known. Intracellular fluxes of NTPs, reduced cofactors, and many other intermediates of metabolic pathways are guaranteed transporters of different endomembranes [45,46]. Chlamydomonas contains one large chloroplast, which locates basic metabolic processes such as photosynthesis, lipid biosynthesis, nitrogen assimilation, glycolysis, and gluconeogenesis. In our study, we discovered the effect of different trophic acclimations on the expression of several transporters providing the exchange of carbon between the chloroplast and cytosol. Acclimation to autotrophic conditions upregulated the expression of *HXT1* and *MEX1* genes encoding transporters responsible for the export of sugars from the plastid (Figure 3 and Figure 7). This data are in agreement with the fact that carbon fixation is a single source of carbon for autotrophic cells. 

Another important function of transporters is the maintenance of reducing power balance [46]. Two main cycles are involved in this process. The first one is C_3_-shuttle [2], which includes triosophosphate transporters (TPT and APE2). Under mixotrophic conditions, the expression of *TPT* is higher in MM cells (Figure 3), which could be a sign of more intensive turnover of energy between the plastid and cytosol. In autotrophic cells, the pattern of the acclimation effect was more complicated (Figure 7). AM cells demonstrated a higher level of *TPT* expression at the beginning of growth and at stationary phase, but this was opposite at the end of culture growth.

The second mechanism for reducing power balancing is a malate valve, which allows for both utilizing excess NADPH in the mitochondria and to supplying the chloroplast metabolism with reducing agents of special importance during adaptation to new conditions [47,48,49,50,51]. Nitrogen regime and central carbon metabolism are probably interconnected tightly in cell recovery [52]. Under mixotrophic conditions, the genes of dicarboxylate transporters OMT1 and DIT showed an opposite reaction to acclimation. *OMT1* was upregulated after autotrophic acclimation, while *DIT* was downregulated. A similar dependence was found at the beginning of growth under autotrophy. However, during culture ageing, alterations were more complicated with mixotrophy. Obtained data allowed for the conclusion that the mechanism of energy balance maintained between plastid and cytosol depends on trophic conditions and is implemented by different transporters. The second substantial function of dicarboxylate transporters is a supply of GS/GOGAT cycle with 2-ketoglutarate [50,53,54]. Thus, a difference in the expression could mirror the specific role of *OMT1* and *DIT,* not only in energy allocation, but also in nitrogen assimilation.

Taken together, acclimation to mixotrophic conditions promotes some redundancy in the synthesis of organic substances. Such an excess could be a result of a more efficient start of photosynthesis at the beginning of culture development. This assumption is confirmed by the fact that, after inoculation, the activity of respiration and photosynthesis sharply increases, and in the stationary phase, it decreases [25,55,56]. Thus, the activation of photosynthesis may depend on the supply of reduced cofactors to the plastid via malate and triose phosphates shuttles [3,47,48,49,50,57]. In our experiments, the higher level of expression for genes encoding dicarboxylate and triose phosphate transporters was observed at the beginning of culture growth. Better energy and carbon supply at the initial stages of growth after mixotrophic acclimation could be explained by the more intense deposition of carbon storage with acetate [5,9,42]. This storage could be mobilized after inoculation and accelerate metabolic restart.

An important question is how intensive the effect of acclimation and how long traits of the previous trophic conditions could be determined after transition? As can be seen in Figure 5 and Figure 9, the effect was more pronounced after mixotrophy, especially at the beginning of the culture growth (Figure 5). Autotrophic acclimation did not show such a clear relationship. Apparently, it is impossible to conclude unequivocally that acclimation always determines further development.

Chlamydomonas cell culture is a very dynamic system. From this, another question is how external factors (trophic acclimation) could modulate it. 

Under mixotrophy, most of the tested genes had similar expression kinetics, regardless of acclimation conditions and had the expression maximum in the middle of the exponential phase, followed by a decrease in culture aging (Figure 4). It can be assumed that such transcript accumulation reflects intensive rearrangements of metabolism, the important feature of which is the direction of the carbon of acetate to the synthesis of lipids [26]. Nevertheless, the profile of expression in the cells of mixotrophic cultures differed according to trophic acclimation. Under MM, the expression level of about a third of the genes was quite high at the very beginning of culture growth and decreased over time. Among those are genes encoding key enzymes of acetate metabolism (ACS1, 2; CIS 2, PCK 1, etc.). This configuration was not similar after acclimation to autotrophy (MA). More genes reached the activation maximum only in the middle of the exponential growth period (MA) (Figure 4). It is possible that such a “delay” in transcript accumulation is the reason for the lag in the growth rate of cultures acclimated to autotrophy. The reflection could be also found in the growth rate: MA cultures reached the density slower than for the MM variant in the second half of the culture development (fourth to sixth DAI). This was accompanied by convergence expression profiles (Table 1, Figure 2B and Figure S3). It can be concluded that after the transition to mixotrophy, cells that had previously developed under autotrophy (MA) conditions did not complete the restructuring of their metabolism for a long time.

In the case of autotrophic cultures, the consequences of acclimation for the dynamics pattern was rather more sophisticated (Figure 8). It should be emphasized that in AM cells, more genes were highly expressed at the beginning of growth (clusters I, III, and V). These genes have a higher level of expression at the beginning of culture development in AM (Figure 7). These genes of ACC subunits and *SBE3* genes indicate a higher level of storage at the beginning of the growth phase in AM (Figure 7). Taken together with higher growth rate, this indicates that acclimation to mixotrophy stimulates photosynthesis at the beginning of growth under autotrophy. In addition, a higher level of expression for genes encoding dicarboxylate and triose phosphate transporters was observed on the first day. These results indicate a high level of energy exchange between the plastid and cytosol. Gradually, the expression of genes associated with the deposition of metabolites and transport decreased (Figure 8), and by the middle of the exponential phase, the level of their mRNA in the cells of AM cultures was not higher than that of AA (Figure 7). The assumption is that the obtained carbon and energy directed in the intensification of growth when the reserves of the previous acclimation period exhausted and deteriorated photosynthesis conditions. AM culture cells were also characterized with a large number of genes with a maximum expression at the end of growth and in the stationary phase compared with the AA variant (Figure 8, clusters IV and V). An increased gene expression at the late stages of development was previously shown in autotrophic Chlamydomonas cultures after changing the lighting mode [43]. It is supposed that an increase in the expression of the genes of primary metabolism, when the growth of the culture is completed or has already been completed, is a sign of the process of adaptation to new conditions—a mechanism for the development of acclimation. 

Note that autotrophic cultures are characterized by a longer period of growth, which is three times longer than that of the mixotrophic ones. Moreover, the final density of autotrophic cultures is twice as low. It could be assumed that the cells were passing less cell cycles. Culture cycle time and number of cell replications are factors that probably affect different regulatory mechanisms, resulting in alterations in dynamics pattern in faster and shorter growing mixotrophic cultures, and slower and longer autotrophic ones. 

## 5. Conclusions and Future Perspectives

The effects of prolongated trophic acclimation on the subsequent development of *C. reinhardtii* batch culture were established. It was shown that mixotrophic pre-cultivation accelerated the growth of cell density in both mixotrophic and autotrophic cultures, especially at the beginning of growth. RT-PCR analysis combined with the methods of multivariate and univariate statics allowed for discovering the significant influence of trophic acclimation on the transcription profile of genes encoding the enzymes of primary metabolism and plastid transporters. The trophic acclimation effect was different in the autotrophic and mixotrophic cultures. In the case of mixotrophic ones, it was stronger and more pronounced at the earlier and middle of the culturing cycle. In autotrophic cells, the effect on transcription level was less severe but more complicated and varied. The differences revealed in the transcriptional profiles’ asymmetry under autotrophy and mixotrophy were concluded to reflect previous metabolic conditions, such as the functioning of photosynthetic machinery, acetate assimilation, storage deposition, and mobilization and reducing power balance.

The advantages of a systemic approach unifying transcriptome, metabolome, and proteome expand the knowledge on the mechanisms of trophic acclimation. Accumulated data might be of use for the elucidation of algae metabolism specification, but also for the acceleration of genetic engineering methods applied for further elevation of algae productivity.

## Figures and Tables

**Table 1:** Parameters of OPLS-DA models for comparing *C. reinhardtii* cultures (mixotrophic and autotrophic) after different acclimation (autotrophy or mixotrophy) at four time points. t1%—proportion of variance associated with the predictive component; N orth. is the number of orthogonal components.

Condition	DAI	Growth Phase		t1%	R^2^X	R^2^Y	Q^2^Y	N orth.
mixotrophy	1	exponential growth	beginning	69	0.898	0.996	0.988	1
	2		middle	88	0.926	0.999	0.99	1
	4		end	36	0.798	0.998	0.853	2
	6	stationary		31	0.722	1	0.858	2
autotrophy	3	exponential growth	beginning	36	0.634	0.997	0.902	1
	6		middle	39	0.61	0.989	0.767	1
	12		end	54	0.836	0.999	0.865	2
	20	stationary		53	0.846	0.997	0.918	2

## Data Availability

Not applicable.

## Supplementary Materials

The following supporting information can be downloaded at: https://www.mdpi.com/article/10.3390/life13061398/s1. Table S1: Genes, products, and primers. Table S2: Cell density. Table S3: Fold changes in relative gene expression and adjusted *p*-value from *t*-test for mixotrophic cultures. Table S4: Fold changes in relative gene expression and adjusted *p*-value from *t*-test for autotrophic cultures. Table S5: *t*-test of differences in PC1 values in pairwise comparison of cultures with different acclimation at each time point (Figures S3 and S4 above). Figure S1: PCA of expression profiles from mixotrophic *C. reinhardtii* cultures. Curves showing the dependence of the explained variance (a) on the number of principal components and the eigenvalues of the components (b), (c) scores plots of the first three principal components. Numbers—time after inoculation, days, color—type of acclimation: green—autotrophy, blue—mixotrophy. Figure S2: PCA of expression profiles from autotrophic *C. reinhardtii* cultures. Curves showing the dependence of the explained variance (a) on the number of principal components and the eigenvalues of the components (b), (c) scores plots of the first four principal components. Numbers—time after inoculation, days, color—type of acclimation: green—autotrophy, blue—mixotrophy. Figure S3: PCA score plots derived from the analysis of the expression profiles from mixotrophic batch cultures of *C. reinhardtii* of four ages acclimated to mixotrophy (blue triangles) and autotrophy (green circles). Samples were taken at 1, 2, 4, and 6 days after inoculation (numbers above plots).
