# Supplementary material for: Effects of Trophic Acclimation on Growth and Expression Profiles of Genes Encoding Enzymes of Primary Metabolism and Plastid Transporters of Chlamydomonas reinhardtii
Source: Life (Basel). 2023 Jun 15;13(6):1398. doi: 10.3390/life13061398 (PMC10301283; doi:10.3390/life13061398)
Supplement: Supplementary file 1 [file life-13-01398-s001.zip › life-2350133-supplementary.pdf]

Table S1. Genes, products, and primers.

Locus tag	Symbol	Product	Primers
CHLRE_10g424500v5	*CPK4*	ribokinase	GCTTCCCGACCTTCTTTG<br>CCGCCCACAATGATGATG
CHLRE_01g026250v5	*AGA1*	alpha-galactosidase	GATGACTGCTGGATGGAGAA<br>TACACGCCGAACTTGAGG
CHLRE_08g359350v5	*CARB1*	acetyl-CoA carboxylase, biotin carboxylase subunit	GCGGCGAGATTGCTGTG<br>GGGATGTTGAGGTAGGACT
CHLRE_04g216950v5	*KASIII*	3-oxoacyl-[acyl-carrier-protein] synthase III	GCCTCCTACGCCAACATCCACAT<br>GCATCACCAGCCAGTCCACAGA
CHLRE_17g709850v5	*ACK2*	acetate kinase	GCGTCTTGGTCCTGAAC<br>ACATTCTCCTTGATGAAATCC
CHLRE_09g396700v5	*ACK1*	acetate kinase	CCTGTCGGGTCGGCAGTG<br>GTGGTCCTTGGCGGGAATCT
CHLRE_12g552200v5	*PHOB*	starch phosphorylase	GTTGTGGAGGGCGACGA<br>TGTGGCAGCGGTGTTCT
CHLRE_01g055408v5	*ACS2*	acetyl CoA synthetase	GCAGGACAAGCAACAGTACGAGG<br>TCCAGGCAGTTGTAGGCGATG
CHLRE_03g181500v5	*STA11*	4-alpha-glucanotransferase	GCTCATCAAGGACGGACTGC<br>ACTGCCACCACGCCAACT
CHLRE_02g080200v5	*TRK1*	transketolase	CGACGCCATCAACAAGTCCAAGT<br>TCGTAGCCAGTCAGGTGCATCAT
CHLRE_12g501000v5	*TPT*	triose phosphate/phosphate translocator	GTCAAGTCGTGGCAGCAGTT<br>GAAGAAGGGCGTCGTGGAG
CHLRE_06g263850v5	*PPT2*	phosphate/phosphoenolpyruvate translocator	GCTCTGCAAAGGCACCAAGTTCAA<br>GCCCTTATCGGACTCGCCATCT
CHLRE_06g307150v5	*AMYB1*	beta-amylase	AGCCAGTGCGTTCTCAGA<br>AAGCGGTCCTCGTCTATCATT
CHLRE_12g519100v5	*ACX2*	acetyl-CoA carboxylase carboxyl transferase subunit alpha	CGGTATCGGCAGCATCAAT<br>ACATCTCACGCAGGTTCAC
CHLRE_17g715250v5	*BCC1*	acetyl-coa biotin carboxyl carrier	CCGAGGAGGTTGAGGCTGCTA<br>CAGGCTGAAACGCTTGCTCTTC
CHLRE_17g713350v5	*OMT1*	oxoglutarate:malate antiporter	GGAGTGCCTGAACAACAAC<br>AGAAGTAGTGCGAGTAGAAGT
CHLRE_02g088600v5	*ACLB1*	ATP citrate lyase, subunit B	CGAGACTCCATCTGTCGCTGCT<br>CTGCGGAAGGAGGCGTAGTTGAT
CHLRE_01g071662v5	*ACS1*	acetyl CoA synthetase	CTGACTCTATTGTTGCGAAGG<br>CGTTGATGTTGGACTGGC
CHLRE_03g149100v5	*CIS2*	citrate synthase	AACACCACCGCCGTCATC<br>TCACATCAATCACCTCCGTCG
CHLRE_02g075700v5*	*RPL19*	ribosomal protein L19	TCGGTTCTGAACTGCGGTCTG<br>CGGGCTGCTTACGGAAGATGA
CHLRE_03g195600v5	*TRE1*	alpha,alpha-trehalase	GACTCCTACTGGGTCATCCTGGG<br>GCTGGCTGCGGTTGGTGTA

CHLRE_03g206800v5	*HXT1*	hexose transporter	CTGATCGGCGTGCTGCTGTTC<br>ACGGAGGATGAGAAGTAGACCAGG<br>G
CHLRE_14g630847v5	*TAL2*	transaldolase	CCGCCTTTCCACCATTGT<br>GCTGTTGCTGTTCTTGCC
CHLRE_05g241850v5	*ACLA1*	ATP citrate lyase, subunit A	TGCCTATCCAGGTGGTTCAGTTCA<br>CGGGCGGTGGCGAAATCAAT
CHLRE_02g141400v5	*PCK1*	phosphoenolpyruvate carboxykinase	TTATGAACAACGCCCGCATCC<br>GCTCAGTCACGCCCATCTCAGT
CHLRE_01g045550v5	*APE2*	triose phosphate translocator	GCTCGTCCGTGCTGTTCTTCA<br>CCTTCTTGTGCTTGTGCTTGCT
CHLRE_12g486600v5	*MEX1*	maltose exporter-like protein	GATGGAAGGGCGACGGACAAC<br>CATTCTGGACGACTTGCGGAAGC
CHLRE_06g260450v5	*DIT*	2-oxoglutarate/malate translocator	GCGGCGACCACCAATGAGAT<br>GCGACTTGATGATGGGCACAAAGA
CHLRE_02g117500v5	*HXK1*	hexokinase	CACTCGGCATCCTCATCCTA<br>CGTCGGCAGCATCATCAT
CHLRE_05g234550v5	*FBA3*	fructose-1,6-bisphosphate aldolase	GCCCTGATGATGAAGTCGTCGG<br>CAACGGTGCCAGCGGTCTT
CHLRE_01g029300v5	*TPIC1*	triose phosphate isomerase	ACGAGGTGGTGGCGAAGAAGACT<br>GGCGTCCAGAACCTTGAACACAGA
CHLRE_02g120100v5	*RBCS1*	ribulose-1,5-bisphosphate carboxylase/oxygenase small subunit 1	CTGAAGCCCGCCGTCAAGG<br>AGAGGAGGCAGGTAGGAGAAGGT
CHLRE_10g444700v5	*SBE3*	starch branching enzyme	CCAACTGCGTCACCATCG<br>GCTTCATCACCTCAATCCACTT

Table S2. Cell density.

Days after inoculation	CD 10^6 cells/mL		t-test
	mixotrophic		
	MM	MA	*p*-value
1	0.031±0.0033	0.012±0.004	0.0031
2	1.1±0.13	0.43±0.14	0.00031
3	4.2±1.2	2.0±0.19	0.03
4	8.6±1.3	6.7±0.54	0.2
5-7 *	9.41±0.95	8.46±0.94	0.27
	autotrophic		
	AM	AA	*p*-value
3	0.15±0.058	0.016±0.0054	0.018
6	0.8±0.21	0.35±0.14	0.016
9	1.9±0.34	1.4±0.21	0.049
12	3.1±0.24	2.9±0.39	0.48
17-23*	4.28±0.54	4.59±0.65	0.27

*stationary phase

Table S3. Fold changes in relative gene expression and adjusted *p*-value from *t*-test for mixotrophic cultures.

	1 days		2 days		4 days		6 days	
**gene**	**FC MM/MA**	**FDR**	**FC MM/MA**	**FDR**	**FC MM/MA**	**FDR**	**FC MM/MA**	**FDR**
CPK4	0.4	0.031	0.26	0.0012	1.42	0.5	0.68	0.52
CARB1	4.49	0.023	0.12	0.096	1.51	0.37	3.83	0.48
KASIII	0.53	0.11	0.37	0.00096	0.25	0.37	1.29	0.65
PHOB	2.04	0.013	0.6	0.011	0.83	0.73	1.1	0.89
ACX2	0.68	0.25	0.13	0.0051	0.27	0.37	0.48	0.48
TRE1	27.16	0.037	0.1	0.0051	1.48	0.37	1.7	0.068
ACK1	1.73	0.16	0.18	0.015	1.29	0.7	0.89	0.71
ACK2	0.43	0.063	0.38	0.027	0.29	0.37	0.49	0.48
ACLA1	0.55	0.077	0.48	0.16	1.03	0.95	0.39	0.28
ACLB1	0.89	0.64	0.6	0.072	1.11	0.9	1.13	0.71
ACS1	8.65	0.022	1.92	0.039	1.16	0.73	1.01	0.99
ACS2	1.73	0.02	0.51	0.015	0.81	0.7	1.13	0.71
AGA1	0.97	0.85	0.05	0.0014	1.67	0.37	0.98	0.92
AMYB1	2.04	0.047	0.15	0.0037	0.68	0.38	0.95	0.86
APE2	0.56	0.057	0.21	0.0051	0.84	0.73	0.88	0.65
BCC1	0.58	0.043	0.14	0.046	0.47	0.37	0.6	0.5
CIS2	2.76	0.015	0.1	0.012	0.87	0.7	1.13	0.71
FBA3	0.5	0.03	0.09	0.012	0.31	0.082	0.83	0.65
HXK1	0.29	0.015	0.37	0.046	0.47	0.7	0.1	0.28
HXT1	0.29	0.03	0.32	0.029	0.78	0.37	0.85	0.65
DIT	6.14	0.016	0.06	0.046	1.56	0.37	1.63	0.52
MEX1	0.37	0.03	0.33	0.0033	0.93	0.37	0.61	0.48
OMT1	0.24	0.019	0.23	0.0012	0.18	0.37	0.54	0.48
PCK1	0.88	0.32	0.19	0.0018	0.26	0.082	1.07	0.86
TPT	2.86	0.022	0.28	0.012	2.1	0.35	1.7	0.5
RBCS1	3.01	0.12	1.67	0.046	5.43	0.37	2.05	0.52
SBE3	1.57	0.015	0.18	0.0072	0.79	0.7	1.21	0.65
STA11	0.78	0.33	0.21	0.0067	0.48	0.37	0.71	0.65
TAL2	5.63	0.016	0.18	0.0087	1.24	0.73	2.83	0.19
TPIC1	1.22	0.38	0.86	0.039	1.14	0.7	0.98	0.95
PPT2	0.84	0.51	0.25	0.033	1.72	0.38	1.34	0.65
TRK1	4.48	0.022	0.35	0.18	0.53	0.7	1.54	0.4

Table S4. Fold changes in relative gene expression and adjusted *p*-value from *t*-test for autotrophic cultures.

	3 days		6 days		12 days		20 days	
**gene**	**FC AM/AA**	**FDR**	**FC AM/AA**	**FDR**	**FC AM/AA**	**FDR**	**FC AM/AA**	**FDR**
CPK4	0	0.12	0.31	0.11	0.57	0.2	0.01	0.15
CARB1	5.23	0.12	1.45	0.67	0.54	0.24	3.02	0.48
KASIII	0.98	0.96	0.78	0.35	1	1	103.11	0.014
PHOB	0.72	0.57	0.84	0.7	0.08	0.18	2.71	0.33
ACX2	1.66	0.77	0.14	0.11	0.26	0.064	6.51	0.0072
TRE1	0.01	0.57	6.02	0.35	31.43	0.064	NA	NA
ACK1	0.75	0.84	0.94	0.91	2.06	0.14	1.41	0.66
ACK2	0.94	0.96	0.78	0.42	1.45	0.36	1.65	0.64
ACLA1	1.04	0.96	0.91	0.9	3.59	0.19	0.94	0.93
ACLB1	1.39	0.77	0.28	0.3	0.72	0.2	2.86	0.42
ACS1	0.18	0.14	0.45	0.38	1.09	0.89	0.62	0.42
ACS2	1.26	0.77	0.89	0.83	0.26	0.12	1.54	0.32
AGA1	3.3	0.12	0.49	0.38	0.04	0.24	9.91	0.071
AMYB1	1.32	0.77	0.84	0.7	0.8	0.25	2.12	0.067
APE2	1.17	0.87	0.72	0.52	1.09	0.3	6.15	0.13
BCC1	2.12	0.57	0.89	0.7	1.81	0.2	1.92	0.5
CIS2	0.95	0.96	0.34	0.19	0.76	0.25	2.12	0.066
FBA3	0.93	0.96	0.66	0.3	1.23	0.44	1.05	0.93
HXK1	0.74	0.77	0.95	0.91	0.74	0.36	2.44	0.46
HXT1	0.26	0.46	0.87	0.7	0.55	0.064	3.56	0.071
DIT	4.31	0.31	0.22	0.0043	0.46	0.36	4.03	0.067
MEX1	1.3	0.8	0.26	0.11	0.93	0.92	3.76	0.41
OMT1	0.77	0.77	0.68	0.52	1.61	0.25	0.58	0.44
PCK1	0.87	0.92	0.42	0.18	0.69	0.25	3.32	0.039
TPT	2.82	0.031	1.09	0.91	0.37	0.11	2.25	0.014
RBCS1	1.01	0.96	1.86	0.35	0.39	0.31	2.75	0.39
SBE3	5.62	0.18	0.34	0.29	0.2	0.064	3.15	0.08
STA11	0.75	0.84	0.37	0.35	0.15	0.25	0	0.014
TAL2	0.44	0.49	0.81	0.73	0.33	0.24	3.87	0.039
TPIC1	1.44	0.37	1.04	0.94	0.38	0.25	2.48	0.24
PPT2	8.57	0.031	1.55	0.35	1.01	1	1.09	0.93
TRK1	1.05	0.96	0.77	0.7	0.4	0.1	2.23	0.42

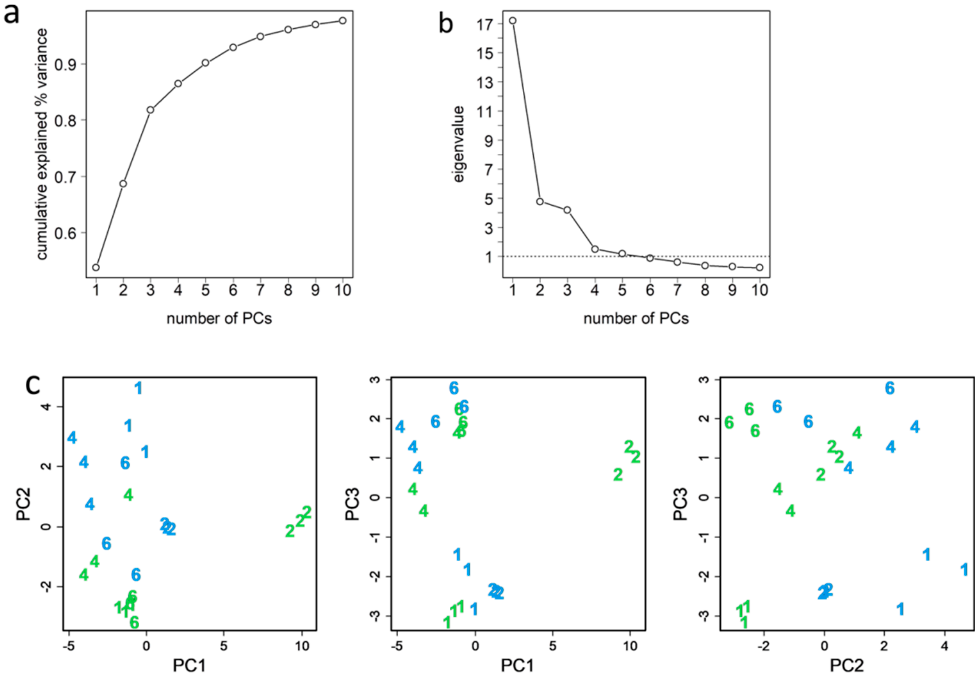

Figure S1. PCA of expression profiles from mixotrophic *C. reinhardtii* cultures. Curves showing the dependence of the explained variance (**a**) on the number of principal components and the eigenvalues of the components (**b**), (**c**) scores plots of the first 3 principal components. Numbers—time after inoculation, days; color—type of acclimation: green indicates autotrophy, blue indicates mixotrophy.

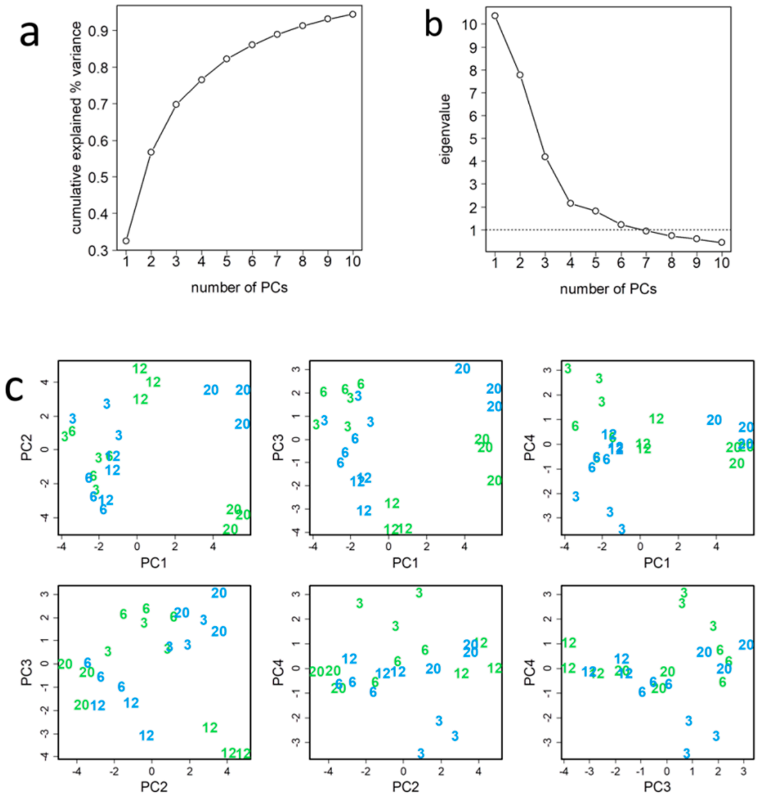

Figure S2. PCA of expression profiles from autotrophic *C. reinhardtii* cultures. Curves showing the dependence of the explained variance (**a**) on the number of principal components and the eigenvalues of the components (**b**),(**c**) scores plots of the first 4 principal components. Numbers—time after inoculation, days; color—type of acclimation: green indicates autotrophy, blue indicates mixotrophy.

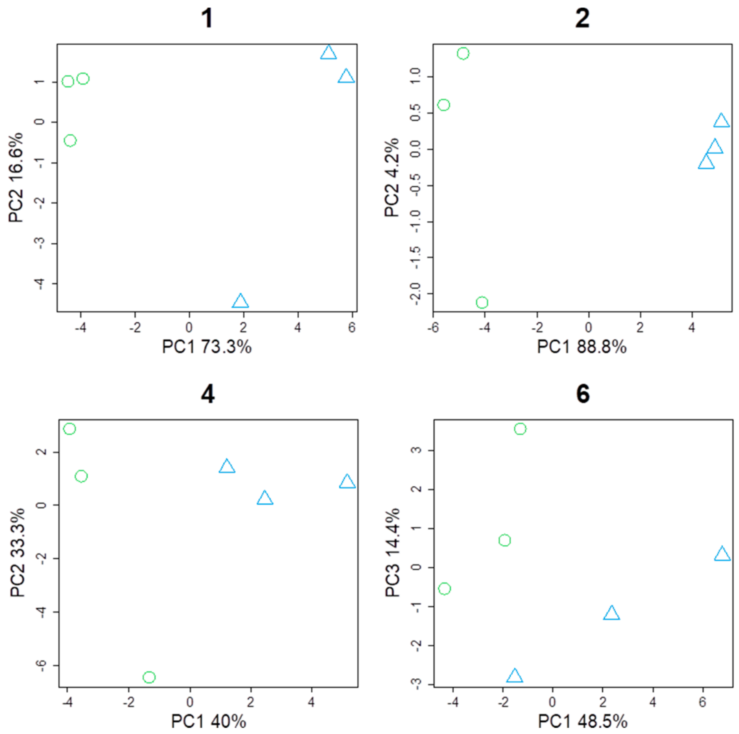

Figure S3. PCA score plots derived from the analysis of the expression profiles from mixotrophic batch cultures of *C. reinhardtii* of four ages acclimated to mixotrophy (blue triangles) and autotrophy (green circles). Samples were taken at 1, 2, 4, and 6 days after inoculation (numbers above plots).

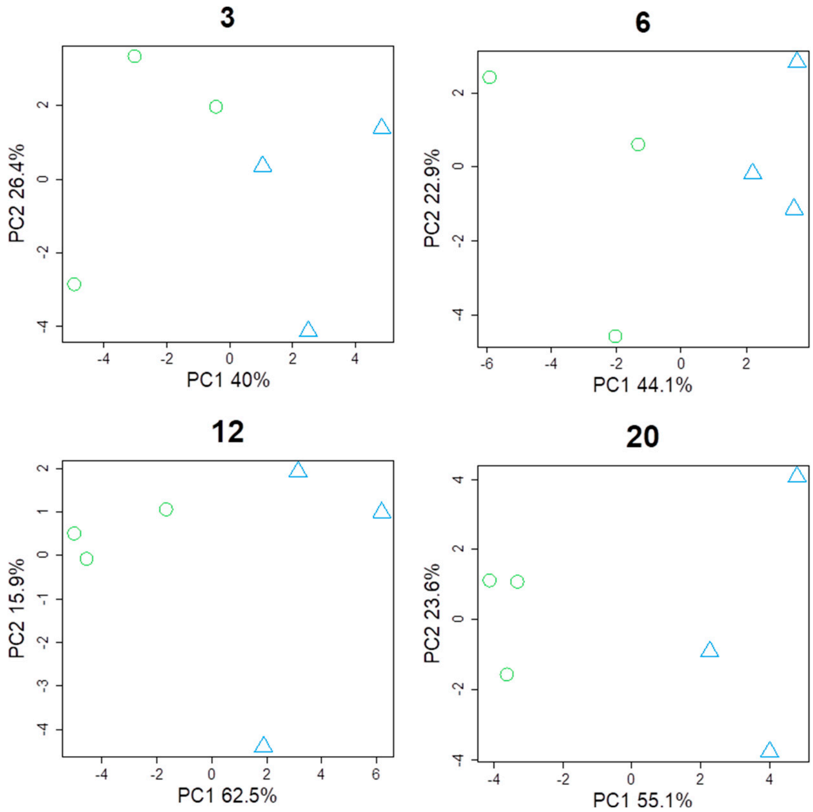

Figure S4. PCA score plots derived from the analysis of the expression profiles from autotrophic batch cultures of *C. reinhardtii* of four ages acclimated to mixotrophy (blue triangles) and autotrophy (green circles). Samples were taken at 3, 6, 12, and 20 days after inoculation (numbers above plots).

Table S5. *t*-test of differences in PC1 values in pairwise comparison of cultures with different acclimation at each time point (Figures S3 and S4 above).

**DAI**	***p***	**fdr**
mixotrophy		
1	0.018	0.029
2	0.00056	0.0045
4	0.018	0.029
6	0.16	0.16
autotrophy		
3	0.032	0.044
6	0.039	0.045
12	0.012	0.029
20	0.0061	0.024
